# Application of Machine Learning and Internet of Things Techniques in Evaluation of English Teaching Effect in Colleges

**DOI:** 10.1155/2022/7643006

**Published:** 2022-03-19

**Authors:** Wen-lan Shang

**Affiliations:** Basic Teaching Department, Huanghe Jiaotong University, Wuzhi, Henan 454950, China

## Abstract

College English classroom teaching evaluation is one of the key issues discussed by all schools at present. First of all, teachers and students are highly concerned about English learning in the compulsory course of English examination, but there are also many problems. The distance network teaching with computer technology as the main body develops rapidly, a large number of video and audio teaching resources network through the network transmission to present in front of learners, through the network transmission of video and audio aided, expand the audience of network teaching, is conducive to the realization of digitalization, information, lifelong, new education goals. Firstly, this article summarizes the problems of poor effect, uneven performance, and mismatch between evaluation and teaching in college English at present. Then, based on the previous works, the author designed a machine learning-based Internet of things technology and verifies its feasibility. The data of teaching experiments show that the writing performance of the students in the lower section of the experimental class is better than that of the students in the middle and high sections. Among them, significant progresses have been made in grammar, length, expression, and structure, which has optimized students' preclass preview efficiency, autonomous learning motivation, quality of homework completion, and afterclass reflection behavior. Finally, the author summarized the shortcomings of this research and puts forward the prospect of relevant research, in order to provide reference value for the national college English blended teaching and promotes the efficient implementation and vigorous development of college English teaching. In the future, the evaluation index system under the pressure of performance can be further studied.

## 1. Introduction

As few decades ago, China listed information technology education as a required course in order to leap-forward the basic education system. Education is an indispensable subsystem of society and deeply affected by social changes [1]. Under the influence of the Internet era, nowadays, teaching methods have undergone earth-shaking changes. The online + offline multichannel English teaching mode represented by hybrid mode has been promoted, which makes the learning resources of learners constantly innovate and develop [2–5]. In recent years, under the impact of the wave of online + offline education, many schools in Beijing, Shanghai, Hunan, Heilongjiang, and other provinces of China have made full use of Internet resources to implement the blended teaching model and achieved considerable results. In the teaching of (listening, to say, read, write) four kinds of ability in English, writing skills is based on the former three, is the highest level of inspection standards, and is the weakest link in English teaching, the hybrid teaching model in English writing teaching practice still gradually exposed some problems, such as precourse reading lack of inspection result in low completion; students' uneven basic level leads to different adaptability of mixed English writing class [6].

Team performance consistent speculation in the English writing guide students' practice. The teaching evaluation of these contradictions are pointing to the hybrid do not mix. Because most of the current hybrid teaching evaluation method have neglected the students' performance in English writing class and teamwork achievements [7, 8], the evaluation of teaching effectiveness has become a hot topic in the field of education. Schools in particular have fully realized that teachers' teaching effectiveness is the core of school survival and development [9]. Although the evaluation criteria of teaching effectiveness are very subjective, they also have a considerable degree of objective laws. More scientific methods and calculate data are applied to make the results more reliable. The evaluation of the teaching effect of college English teachers is also helpful for administrators to have a comprehensive understanding of the teaching situation of college English teachers. For example, college English teachers improve classroom teaching methods by new courses and pay attention to the interaction with students. The usage of modern teaching media is conducive to improving the teaching effect. It is a difficult problem with great research value to accurately grasp and provide scientific standards for the school to formulate the direction of subject development and teacher planning to comprehensively study the classroom teaching effects [10–13]. However, the Internet of Things technology is not involved in the above works.

Recently, the Internet of things technology has attracted a lot of attention, and various assessment systems and methods have been formulated to promote the establishment of good political quality, noble moral sentiment, and strong working ability of English teachers [14]. However, the evaluation of classroom teaching effects of college English teachers involves many aspects and factors. The knowledge level, cognitive ability, and personal preference of the participants can directly affect the evaluation indicators. It is difficult to exclude the influence brought by individual factors, and the obvious fuzzy characteristics of evaluation indicators also cause difficulties for the evaluator in specific operation [15, 16]. In addition, in the past teaching evaluation, the qualitative analysis method is often used, which ignores the factors existing in itself [16, 17].

The theoretical significance mainly includes two points: first, it analyzes the English classroom teaching model and then applies the fuzzy comprehensive evaluation method in the field of middle school English classroom teaching evaluation [18]. According to the uniqueness of the middle school English classroom assessment, after the description and analysis of the factors of student evaluation of expert evaluation and teacher evaluation factors, the weights are applied to teaching appraisal, to further examine the rationality of the framework of middle school English classroom teaching evaluation. Based on the fuzzy comprehensive evaluation method, this article presents a sample of English teaching evaluation in middle school [19, 20].

It is conducive to the in-depth discussion on the evaluation of middle school English classroom teaching effect. Second, a scientific, effective, and reasonable evaluation model system of classroom teaching effect of Chinese and English is put forward to build high-quality and efficient teaching staff [21]. Third, the machine learning evaluation method and Internet of things technology described in this article can be used for reference in other courses, which can be further studied. Fourth, in the practice process, school leaders can well cooperate with English classroom teaching practice [22], but the evaluation of English classroom teaching effect needs to be adjusted because leading teachers are faced with the demand of performance assessment [23].

The main contributions of this article are listed below:

1. From the above analysis, we know that the above methods have studied the English teaching effect in colleges widely. However, some problem still exists.
2. For example, no scholar has applied the machine learning-based Internet of things technology to this field till now, so the research here is still a blank, which has great theoretical research and practical application value for logistics enterprises.

This article mainly contains five parts. The first section gives the research background and significance. The second section gives the related work in the field. The third part is the evaluation of English teaching effect by computer Internet of things. The fourth part gives the simulation results to demonstrate the superiority of all methods. At last, the fifth part is the conclusion of this article.

## 2. Related Work

Speckesser et al. [24] proposed a smart classroom learning media library based on mobile technology. Farid et al. [25] analyzed relevant theoretical and practical questions from three aspects: the concept and application of information technology education. Specifically, they mainly answered basic questions such as what is a smart classroom, how to organize and construct it, and how to apply it to the classroom. Intelligent classroom brought to the attention of the world wisdom class system in the world to develop, to further expand the wisdom classroom environment, and to realize the diversification of education and intelligent. Singapore announced in 2016 the overall plan of the intelligent state, which will be wise in the planning of education as the key elements of the select few pilot schools. Australia and South Korea also have their own smart education programs. Ozan and Kincal [26] proposed that preparation for college English teaching is effectiveness research in teaching, and this teaching method is not linked with students' achievements. Giraldo [27] thought education is very important for the people in his whole life, he bound on the education to the children now extremely discontent, think should with interest as the king, to adapt to in their own conditions, to students, activities, experience, the three as the core, to replace the classroom. The concept of English teachers and books and his attitude towards classroom teaching evaluation effectively reflect that in the process of classroom teaching evaluation in middle schools, students are not only the components of classroom teaching but also the soul and should be encouraged to take the initiative to participate evaluation.

Based on previous studies, Limber et al. [28] found that evaluation has great impact on both teaching, writing, and students' learning of writing by observing and investigating undergraduates' opinions on writing tests and their reflections after taking writing classes. Klimova [29] found the shortcomings of line evaluation and carried out reforms to further refine the evaluation criteria, including the validity of logical points of discourse, persuasive arguments, and other factors into the evaluation. Through empirical research on more than 100 English compositions, they verified the reliability of this evaluation method. Because the evaluation method of examination does not directly promote the level of English thinking and logical argumentation of many learners, the range of applications will be limited. Masats and Guerrero [30] critically examined and found that the automatic evaluation system was not perfect either because some students were not proficient enough to effectively evaluate each other's articles in online mutual evaluation, and their attention was also focused on the language level as teachers' evaluation. Almanthari et al. [31] found through the follow-up study of teacher teaching that the application of technology in mathematics teaching produced good teaching effects. The effective application of technology tools such as interactive whiteboard not only improved teachers' information teaching ability but also improved students' ability of expression, operation, and understanding of subject concepts [32, 33].

This article expounds the information technology, and curriculum integration not only should focus on students' view and main body status but also should pay attention to the teacher in the teaching. Therefore, in the research process of teaching design, we should attach importance to it. Guiding, enlightening, and supervising professors can help students to master knowledge and skills systematically and make the knowledge and skills acquired by students more scientific and systematic. In terms of the proportion of research results, there are more research results in science and engineering related to information technology, curriculum integration concept, and specific subject content integration, although there are relatively few research in humanities and there are even fewer research results in College English [34]. Among them, many domestic scholars especially puts forward the concept of information technology and curriculum integration of guiding the teaching design and the important role in the whole teaching, namely, each discipline has unique characteristics, blind Wang universality of general technology already outdated and not feasible, and should combine the characteristics of the actual specific subjects to integrate information technology through the design method and the whole teaching process [35]. At present, the research trend and path of information technology and curriculum integration concept turn to micro to solve the practical problems of teaching; however, there are still some problems and limitations in domestic research. For example, relevant researchers focus on preservice and primary and secondary school teaching, whereas the research on university teaching has not been widely popularized and deepened. In actual teaching, the integration of information technology and specific subject content is not ideal, and the teaching practice with deep integration is lacking. Relevant studies show that teachers should plan and design the curriculum of integrated technology, that is, apply the idea of information technology and curriculum integration to optimize the teaching to enhance the teaching effect and promote students' learning ability [36].

## 3. Evaluation of English Teaching Effect in Internet of Things

### 3.1. The Process of English Teaching Effect Evaluation

**Figure 1:**
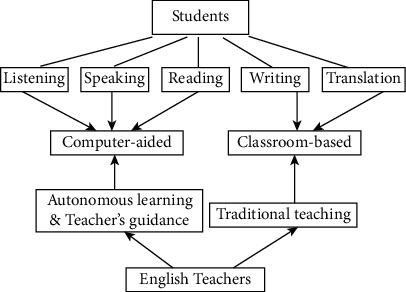
The whole system framework.

The multimedia teaching platform system based on streaming media technology should have the following functions [37]: (1) User login function, including user login and user information viewing and modifying; (2) system instructions, including resource index classification and query function description; (3) video information index function; (4) video information retrieval function, including search by chapter, search by knowledge point, search by subject; and (5) video information playback function. After the user enters the platform system, he or she first appears in front of the user login page, requiring users to enter your user name and password, according to the user input user name and password; the system will enter the backstage supporter's database to check for comparison and determine the user's identity is a system administrator or learner, if learners are learning into the interface. The whole system of the method is given in Figure 1. In this system, an Internet of thing framework is used to monitor and control the whole system of English teaching. In this situation, all data and status can be stored into a cloud platform, and we can transfer them using Internet of things. Meanwhile, in order to implement an intelligent way, a machine learning model named fuzzy comprehensive evaluation is bedded into this system, and it can process and analyze the collected data. Then, it can return the analyzed data to users.

In the implementation process of college English classroom teaching evaluation, the evaluation results of professional students and teachers are calculated according to the collected data and then obtained by comprehensive calculation. Then, the scoring method is taken as comparison. The implementation results show that the evaluation method of English teaching effect described in this article can be very effective to meet the needs of English teachers' classroom teaching evaluation. The author collected the data in the teaching experiment process, used SPSS software for data analysis, and sorted out the questionnaire, interview records of students, and the research conclusion of this article. The last part is the conclusion reference appendix and the acknowledgements. It is hoped that the learning effect evaluation index system in this study can enhance students' enthusiasm and interest in English writing, promote students to improve their learning efficiency in English writing class, and make contributions to relevant research fields in China.

### 3.2. Fuzzy Comprehensive Evaluation Method

Fuzzy comprehensive evaluation as one of the machine learning models is the evaluation of something using fuzzy mathematics tools, evaluation index weights, and evaluation sets; generally, it can be divided into the very satisfied, satisfied, not satisfied, and very dissatisfied with four levels, then the weight of each variable in the clear degree of the vector, and belongs to, using the method of fuzzy evaluation matrix, finally finish the matrix and weight vector of fuzzy processing and complete normalized operation. The results of fuzzy comprehensive evaluation are obtained, and a comprehensive evaluation model is constructed as follows:

Determine the evaluation index weight set *C* of the evaluation object:

(1) $$\begin{array}{c} U=\left(U_{1},U_{2},\ldots ,U_{n}\right). \end{array}$$

Determine the judgment set *V*:

(2) $$\begin{array}{c} V=\left(V_{1},V_{2},\ldots ,V_{m}\right). \end{array}$$

To establish the fuzzy relation matrix *R*, after constructing the fuzzy subset, each evaluation factor should be quantified to determine the subordination degree of each fuzzy subset, so the fuzzy relation matrix formula is obtained as follows:

(3) $$\begin{array}{c} R=\left[\begin{array}{cccc} r_{11} & r_{12} & \cdots & r_{1m}\\ r_{21} & r_{22} & \cdots & r_{2m}\\ \cdots & \cdots & \cdots & \cdots \\ r_{n1} & r_{n2} & \cdots & r_{nm} \end{array}\right]. \end{array}$$

Fuzzy comprehensive evaluation method uses analytic hierarchy process to determine the weight vector of evaluation factors:

(4) $$\begin{array}{c} W=\left(W_{1},W_{2},\ldots ,W_{n}\right). \end{array}$$

The commonly used method is the principle of maximum membership degree, but the disadvantages of this method are relatively reluctant, and the information will be lost a lot, which will lead to the deviation of the evaluation results. Therefore, the weighted average method is proposed to obtain the membership degree method, and the ranking of several evaluated things, so this article adopts the weighted average model, Finally, *W* and *R* are combined to obtain the result vector *B*:

(5) $$\begin{array}{c} B=C^{{}^{\circ}}R=\left\{C_{1},C_{2},\ldots ,C_{n}\right\}{}^{\circ}\left[\begin{array}{cccc} r_{12} & \cdots & r_{1m}\\ r_{21} & r_{22} & \cdots & r_{2m}\\ \cdots & \cdots & \cdots & \cdots \\ r_{n1} & r_{n2} & \cdots & r_{nm} \end{array}\right], \end{array}$$

(6) $$\begin{array}{c} \chi \left(i,j\right)=0\text{ or }1\text{,} \end{array}$$

In addition, the sum of the weights occupied by students and teachers in the whole goal can also be obtained by analogy, so the publicity is satisfied:

(7) $$\begin{array}{c} C_{1}+C_{2}+\cdots +C_{n}=1. \end{array}$$

The weight of each first-level indicator of expert students and teachers in college English classroom teaching is the sum of second-level indicators, so all of them meet the calculation of expert students and teachers' indicators:

(8) $$\begin{array}{c} C_{i}=C_{i1}+C_{i2}+\cdots +C_{ik_{i}}\left(i=1,2,\ldots ,n\right), \end{array}$$

(9) $$\begin{array}{c} \left\{\begin{array}{c} C_{1}=C_{11}+C_{12}+\cdots +C_{1k_{1}},\\ C_{2}=C_{21}+C_{22}+\cdots +C_{2k_{2}},\\ \cdots \\ C_{n}=C_{n1}+C_{n2}+\cdots +C_{nk_{n}}. \end{array}\right. \end{array}$$

Calculate the total score of the questionnaire of English classroom teaching evaluation in middle school. Before calculating the weight of second-level indicators of experts, the total score of the questionnaire of experts, students, and teachers needs to be calculated. Now we know the score of second-level indicators, the score of first-level indicators is the sum of all second-level indicators under the branches of the first-level indicators, and the total score *F* is the sum of the scores of all first-level indicators. Therefore, it is assumed that expert student teachers have *N* first-level indicators, and the number of second-level indicators in the subbranch of each first-level indicator is the score of the *i*-th first-level indicator, and is given by the following formula [38]:

(10) $$\begin{array}{c} F_{i}=F_{i1}+F_{i2}+\cdots +F_{ik}\left(i=1,2,\ldots ,n\right). \end{array}$$

The total score *F* of the second-level index of expert students and teachers satisfies the following formula, respectively:

(11) $$\begin{array}{c} \left\{\begin{array}{c} F=F_{1}+F_{2}+\cdots +F_{n},\\ F=F_{11}+F_{12}+\cdots +F_{1k_{1}}+F_{21}+F_{22}+\cdots +F_{2k_{2}}+\cdots +F_{n1}+F_{n2}+\cdots +F_{nk_{n}}. \end{array}\right. \end{array}$$

**Figure 2:**
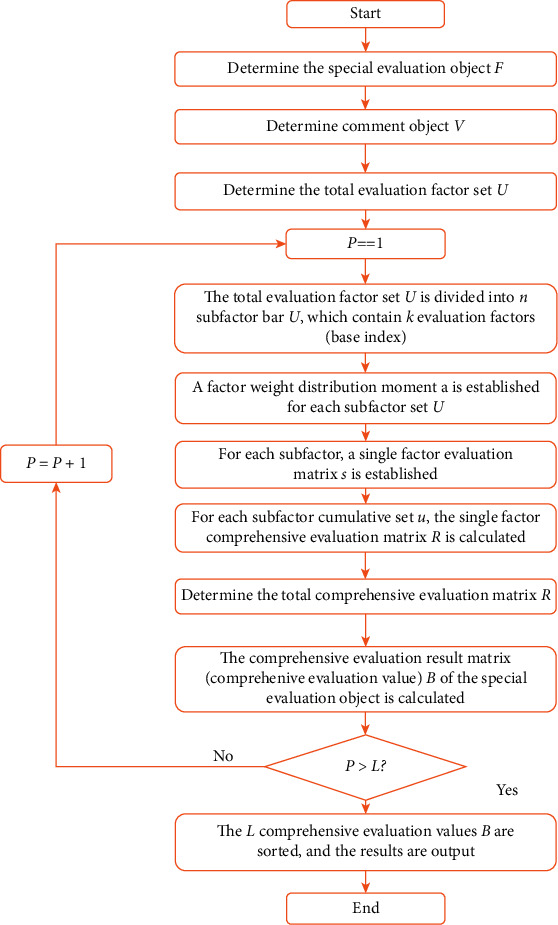
The schematic diagram of evaluation of English teaching effect.

The schematic diagram of a typical computer Internet of things for Evaluation of English teaching effect is shown in Figure 2.

## 4. Experimental Results and Analysis

### 4.1. Introduction to Experimental Environment and Data Set

Before the experiment, the author selected two classes with similar English writing skills as the experimental class and the control class by referring to the previous test results of the school. Before the experiment, the author conducted a writing pretest on the two classes. After collecting the score data, the author calculated the average sum of the two classes and conducted an independent sample *T* test. According to the independent sample *T*-test results, the significance level value is 0.968, which is greater than 0.05, indicating that there is no significant difference in English writing level between the two classes. Therefore, these two classes meet the requirements and can be used as experimental objects to participate in this teaching experiment. Therefore, the author implemented the mixed teaching arrangement of the two classes as planned and supervised all variables at all times to ensure the scientific and fairness of the experiment.

### 4.2. Experimental Results Analysis

**Figure 3:**
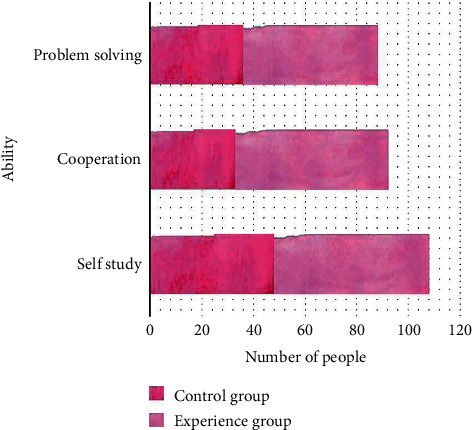
Evaluation of English ability of different number of college students.

In this study, a total of 60 students of software were chosen as research samples. There were 37 male students and 23 female students, in order to ensure the scientific aspect and accuracy of the research experiment; a scientific pre-experiment survey was carried out before the implementation of this study. After research and implementation, the revised scale of Chinese English listening and speaking ability was used as a measuring tool to evaluate students in university in the early stage. The data were collected in three stages and lasted for three months. The changes of the three groups of experimental data were compared with the preliminary initial data, the midterm experimental data, and the later experimental data to obtain the research results. The data software SPSS22.0 was used as the data statistics and analysis tool to measure and evaluate whether the English listening and speaking ability of college students was improved in the early and middle periods. The specific results are shown in Figure 3.

**Figure 4:**
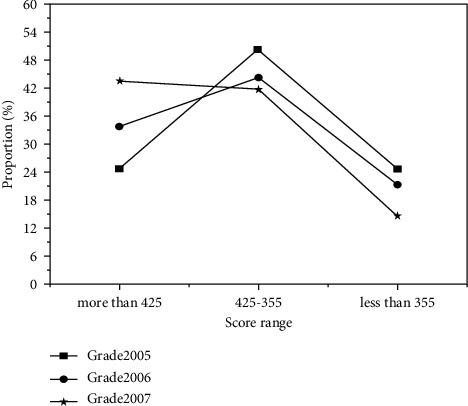
The score range comparison.

Secondly, the research on the equal scope of college students, statistics, and analysis found that the teaching form of teaching context setting, students' preferences, and acceptance are not the same. Finally, the teaching adjustment in the research period was carried out, and video dubbing and role-playing were adopted as the main setting methods according to students' learning needs and interests, which improved students' enthusiasm and initiative in learning. Teaching adjustment in the middle of the study fits students' learning needs and interests, and remarkable teaching results have been achieved in the later stage of the study. The specific results are shown in Figure 4.

**Figure 5:**
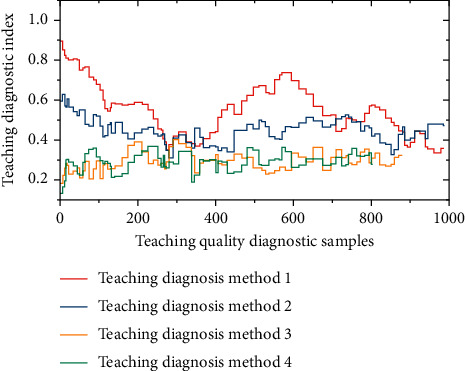
Improvement trend of teaching diagnosis indicators of college students.

Finally, the improvement trend of teaching diagnosis indicators of college students is plotted as shown in Figure 5. From the middle of the early stage to the late stage, the maximum value (maximum and minimum value) has a certain degree of improvement. From the perspective of the mean value, the score of college students' listening and speaking ability ranges from 0.132 to 1.176 in the middle and late stages of the study, and the standard deviation and variance values are within a reasonable range, indicating that students' English listening and speaking ability has been improved.

**Figure 6:**
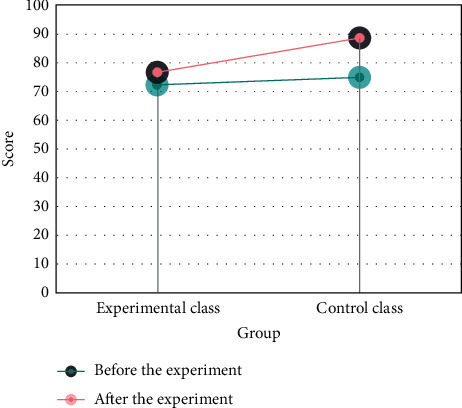
English listening ability score result.

This study also used descriptive statistical analysis and then to study research this paired *T* test, based on the results of statistical analysis data to verify whether the students' English listening ability score improved, according to previous research data to the student to do descriptive statistics analysis results; the table is shown in Figure 6, the early more than average. The maximum value is 87.8, and the minimum value is 76.9. On the whole, the students' English listening and speaking ability is slightly worse than the critical value, so the scoring ability needs to be improved.

**Figure 7:**
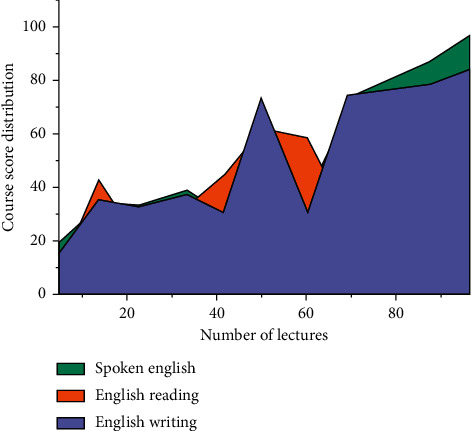
Distribution of score data in English teaching effect.

As shown in Figure 7, the distribution of score data in English teaching effect shows that with the increase of class hours, no-matter English listening, reading, and writing ability have been improved to different degrees, but the degree of improvement of different abilities is different. It shows that with the increase in the amount of practice, the English teaching effect in colleges and universities will eventually be improved.

**Figure 8:**
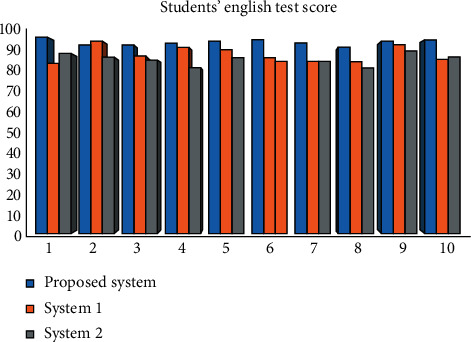
English test scores of college students under different systems.

In Figure 8, the method proposed in this article obtained the best test scores, indicating that both students and teachers take the evaluation scales seriously and organize the learning effect of each lesson. English composition data put together into draft formed personal electronic files from the teachers' level, which reduces our homework originally used to have a lot of time, greatly reduces the pressure of work at the same time, and saves time review supplement and the root of the machine, according to the teaching platform data, to evaluate the students' self-study before class and after class and to supervise the whole learning process of students. It can be seen that the implementation of the blended learning effect evaluation index system in high school English writing classes has reached the expected value.

## 5. Conclusions

In the research of this article, the classroom teaching effect is concerned with teachers' teaching methods and attitudes, teachers' communication ability, and students' independent learning ability. In the design process of the article, it is more necessary to improve these capabilities through a large number of relevant literature review, learning the model algorithm; the fuzzy comprehensive evaluation method used in this article to evaluate the model calculation process is more cumbersome but easy. It can provide a new evaluation idea for the evaluation of English classroom teaching effect in middle schools and can also be used in other aspects of evaluation problems. In the creation process of this article, the following aspects are emphasized. The author first issued investigation to students' questionnaire in order to understand the student existing opinion and the suggestion, the existing evaluation system for the design and establishment of high school student's English learning effect evaluation index system for the basis. Then, the author, in turn, investigated the experimental classes, and after that, semistructured interview was carried out on the students and the teacher, to test the validity of the evaluation index system.

English listening and speaking ability is an individual's long-term application ability, which should be accumulated and studied in sufficient time. Due to the special situation this year, the preliminary investigation and formal implementation of the study only lasted three months, which may have a slight impact on the accuracy of the measurement of college students' English listening and speaking ability. There may be some problems such as insignificant data or insignificant significance, which may have a certain impact on the research conclusion. There are some ontological problems in the measurement dimensions of the Chinese English language listening and speaking ability scale, that is, the incompatibility with the actual teaching in colleges and universities. After the revision of the initial scale, such problems have been solved to some extent. However, the degree of perfection of the revised scale is not very sufficient, including some practical problems. For example, the English cultural factors hidden behind the questions are not fully reflected, and sufficient language scene description and hypothesis are not provided.

Because the English knowledge structure and learning style of college students are different, it is difficult to ensure that the English listening and speaking ability of each college student can be significantly improved in this study. The optimization design of university teaching from the perspective of integration needs further research and exploration in order to adapt to and solve the practical problems of university teaching and student learning. By carrying out relevant studies on the evaluation system of blended learning effect, the author has gained a further understanding of the evaluation research, as well as a deeper perception of the teaching practice and has the following prospects for subsequent studies on the evaluation of blended learning effect: The blended teaching mode has become an inevitable trend of education, but the evaluation research on blended teaching is still relatively scarce. It is hoped that the research on the effectiveness of blended evaluation can be gradually improved in the future research to provide theoretical guidance for teaching practice.

## Data Availability

The data used to support the findings of this study are available from the corresponding author upon request.
